# Establishment and application of novel culture methods in *Marchantia polymorpha*: persistent tip growth is required for substrate penetration by rhizoids

**DOI:** 10.1080/19420889.2022.2095137

**Published:** 2022-07-06

**Authors:** Hikari Mase, Hirofumi Nakagami, Takashi Okamoto, Taku Takahashi, Hiroyasu Motose

**Affiliations:** a Graduate School of Natural Science and Technology, Okayama University, Okayama, Japan; b Protein Mass Spectrometry, Max Planck Institute for Plant Breeding Research, Cologne, Germany

**Keywords:** Rhizoid, tip growth, NIMA-related kinase, liverwort, *Marchantia polymorpha*

## Abstract

A NIMA-related protein kinase, MpNEK1, directs tip growth of rhizoids through microtubule depolymerization in a liverwort *Marchantia polymorpha*. The Mp*nek1* knockouts were shown to develop curly and spiral rhizoids due to the fluctuated direction of growth. Still, physiological roles and mechanisms of MpNEK1-dependent rhizoid tip growth remain to be clarified. Here, we developed novel culture methods to further study rhizoid growth of *M. polymorpha*, in which plants were grown on vertical plates. We applied the established methods to investigate MpNEK1 function in rhizoid growth. Rhizoids of the wild-type and Mp*nek1* plants grew toward the gravity. The aerial rhizoids were longer in Mp*nek1* than in the wild type. When the rhizoids were grown on the surface of a cellophane sheet, rhizoid length was comparable between the wild type and Mp*nek1*, whereas Mp*nek1* developed more rhizoids compared to the wild type. We also applied gellan gum, which is more transparent than agar, to analyze rhizoids grown in the medium. Rhizoids of Mp*nek1* displayed defect on entering into the solid medium. These results suggest that Mp*nek1* rhizoids have the deficiency in invasive tip growth. Thus, stable directional growth is important for rhizoids to get into the soil to anchor plant body and to adsorb water and nutrients. Collectively, our newly designed growth systems are valuable for analyzing rhizoid growth.

## Main text

Tip-growing filamentous cells, such as root hairs and rhizoids, play pivotal roles in nutrient uptake from and anchorage to the soil in plants. Microtubules and actin filaments have been shown to regulate tip growth of root hairs and rhizoids [1,2]. In a liverwort *Marchantia polymorpha*, NIMA-related protein kinase, MpNEK1, stabilizes growth direction of rhizoids through microtubule depolymerization in the apical dome [3]. The MpNEK1 knockouts exhibit highly crooked rhizoids, in which growth direction is frequently changed over time. Another microtubule regulator, WAVE DANPENED-LIKE (MpWDL), also regulates tip growth of rhizoids by stabilizing longitudinal microtubules in the shank region of the rhizoids of *M. polymorpha* [4,5]. Rhizoid is an emerging model of tip growth, and few studies have been conducted on it. Although rhizoid differentiation and growth were studied by various culture methods and live imaging [3,5–8], further development of experimental systems should be helpful. In this study, we established culture methods and analyzed rhizoid growth of the wild type and Mp*nek1* knockouts.

**Figure 1.**
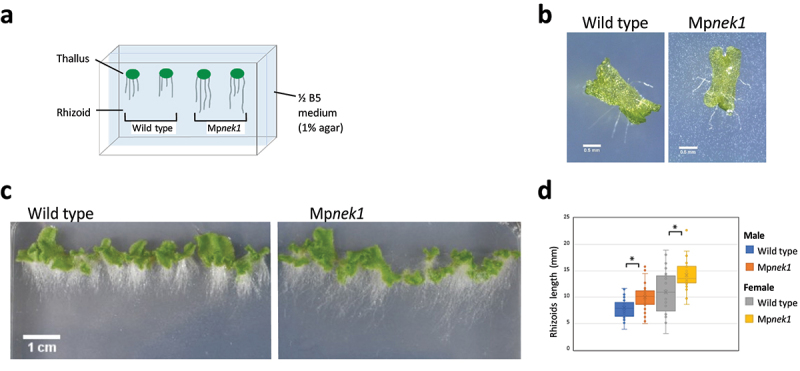
Rhizoid growth in the vertical culture method. (a) Vertical culture method. Gemmalings are grown vertically in a culture dish filled with ½ B5 medium (1% agar). Rhizoids grown over the surface of medium are examined. (b) Four-day old gemmalings of the wild type and Mp*nek1* mutant. Photographed with a stereomicroscope S8APO0 (Leica Microsystems) equipped with a CCD camera DFC500 (Leica). (c) Sixteen-day old wild type and Mp*nek1* mutant, photographed with a digital single-lens reflex camera (Nikon D5600). (d) Quantification of the rhizoid lengths of the wild type and Mp*nek1* mutant (Male wild type: Tak-1, Female wild type: Tak-2, Male Mp*nek1* mutant: line #2-4, Female Mp*nek1* mutant: line #43). Values are shown by box plots (n = 26–47). Asterisks indicate significant difference compared with the wild type (Student's *t*-test, *P < 0.05).

First, we analyzed rhizoid growth of the wild type and Mp*nek1* knockouts in vertical culture method (Figure 1). Gemma, asexual propagule, was placed on the half-strength B5 agar medium and cultured vertically (Figure 1(a)). In this method, rhizoids growing on the agar medium or in the air (aerial rhizoids) were clearly observed as the white filamentous structures, allowing us to easily analyze the morphology and length of the rhizoids. In both the wild type and Mp*nek1*, rhizoids grew to the gravity vector (Figure 1(c)). Our quantification of randomly selected aerial rhizoids showed that the length of the rhizoids was significantly longer in the Mp*nek1* knockouts than in the wild type (Figure 1(d)). This result indicates that the Mp*nek1* knockouts develop longer aerial rhizoids compared to the wild type.

**Figure 2.**
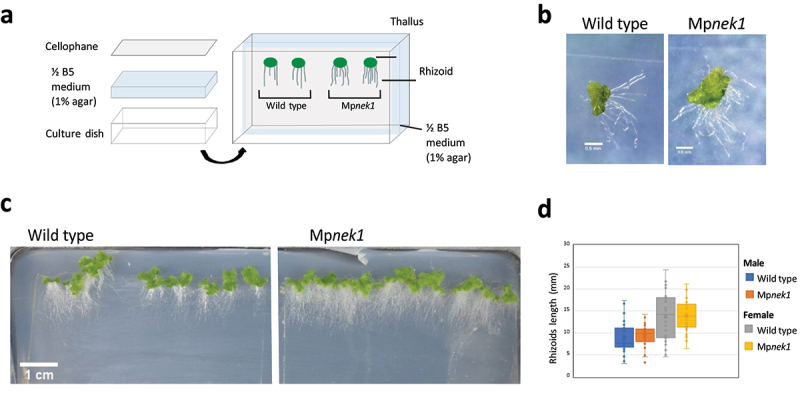
Rhizoid growth in the cellophane culture method. (a) Cellophane culture method. Gemmalings are grown vertically on a cellophane sheet placed on the surface of a ½ B5 medium (1% agar). The cellophane sheet hinders rhizoids from getting into the medium. Rhizoids grown on the surface of medium are examined. (b) Four-day old gemmalings of the wild type and Mp*nek1* mutant. Photographed with a stereomicroscope S8APO0 (Leica Microsystems) equipped with a CCD camera DFC500 (Leica). (c) Sixteen-day old wild type and Mp*nek1* mutant, photographed with a digital single-lens reflex camera (Nikon D5600). (d) Quantification of the rhizoid lengths in the wild type and Mp*nek1* mutant (Male wild type: Tak-1, Female wild type: Tak-2, Male Mp*nek1* mutant: line #2-4, Female Mp*nek1* mutant: line #43). Values are shown by box plots (n = 40). No significant difference is observed (Student's *t*-test, P > 0.05).

We consider a possibility that the above method was not appropriate to estimate rhizoid length because rhizoids grown in the medium could not be observed, resulting in the underestimation or overestimation of rhizoid length. To examine this, we developed cellophane culture method (Figure 2). Gemmalings were grown vertically on a cellophane membrane placed on the half-strength B5 agar medium (Figure 2(a,b)). The cellophane membrane is an obstacle hindering rhizoids from growing into the medium, so that all rhizoids were grown on the cellophane sheet. The length of the rhizoids was assessed after 16 days in the wild type and Mp*nek1*. There was no significant difference in the rhizoid length between the wild type and Mp*nek1* (Figure 2(c,d)). Thus, the growth ability of rhizoids might be identical between the wild type and Mp*nek1*. The longer rhizoids of Mp*nek1* in the previous method could be attributed to the defect of penetration into the medium (relatively increased aerial growth in appearance). This is consistent with the previous study, showing the same rhizoid length in juvenile gemmalings between the wild type and Mp*nek1* [3].

**Figure 3.**
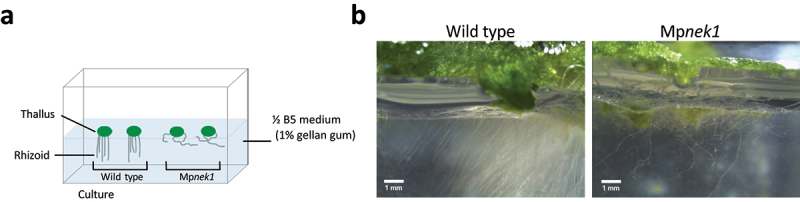
Rhizoid growth in the gellan-gum culture method. (a) Gellan-gum culture method. Gemmalings are grown on the ½ B5 medium solidified in the bottom half of a square culture dish by 1% gellan gum, which is more transparent than 1% agar and is suitable for lateral observation. Rhizoids grown inside the medium are examined. (b) Rhizoids in the 29-day-old of wild type and Mp*nek1* mutant. Photographed with a stereomicroscope S8APO0 (Leica Microsystems) equipped with a CCD camera DFC500 (Leica).

To clearly visualize rhizoids grown in the medium, we applied gellan gum, which is more transparent than the agar medium (Figure 3). Gemmalings were grown on the half-strength B5 medium solidified by 1% gellan gum in the bottom half of the square culture dish (Figure 3(a)). In this method, it is easy to observe rhizoids in the medium from the side view. Most rhizoids of Mp*nek1* failed to penetrate into the medium and rather grew over the medium (Figure 3(b)), whereas the wild type rhizoids entered into the medium. Thus, the Mp*nek1* rhizoids have reduced competency of substrate penetration.

Collectively, our results demonstrate that Mp*nek1* rhizoids have the deficiency in invasive growth. Since MpNEK1 has been shown to direct tip growth of rhizoids [3], MpNEK1-mediated stabilization of growth direction might be required for the penetration of rhizoids into the solidified medium. Persistent tip growth through the stabilization of a growth site may increase the penetration force of rhizoids to promote the anchorage of plant body and adsorption of water and nutrients from the soil.

It would be noteworthy that the Mp*nek1* mutants have more rhizoids than the wild type (Figure 2(b,c)). MpNEK1 may suppress the excessive formation of rhizoids. However, MpNEK1 rather direct tip growth of rhizoids. Therefore, it is more feasible that formation of abnormal rhizoids (i.e. curly, twisting rhizoids in Mp*nek1*) may activate quality surveillance mechanism to compensate for the defect of rhizoids by promoting new rhizoid formation.

Here, we developed new culture methods, which are useful for analyzing rhizoid growth. Especially, cellophane culture method could be applicable to various physiological and biochemical experiments. It is easy to transfer plants from one medium to another medium. Further, this method is less stressful for plants in the sample isolation and transfer experiments. In the usual culture on the agar medium, it is difficult to pick up plants from the medium without any damage because rhizoids firmly hold the medium and anchor plant body.

In addition, cellophane culture can be adapted to the study of responses to various stresses. Rhizoids experience different physiological effects when growing on the cellophane sheet. Embedded in the agar medium, rhizoids are exposed to various stresses including compression, friction, osmotic stress, and hypoxia, while they are relatively free from the environmental stresses on a cellophane membrane. On the other hand, friction could occur between the cellophane sheet and rhizoids. In fact, 4-day-old wild-type rhizoids showed wavy morphology on the surface of cellophane sheet (Figure 2(b)). This could be attributed to the response of rhizoids to contact stimuli. As a side note, the size of thalli is slightly smaller in the cellophane culture than in the usual agar culture without cellophane. It may be due to inefficient absorption of water and nutrients from the medium, implying negative effect of cellophane on adsorption and/or the absorbing function of rhizoids.

In summary, new culture methods demonstrate essential function of MpNEK1-driven tip growth in the substrate penetration by rhizoids. Our novel methods could be useful for various experimental approaches.
